# Supraglottic Airway Device in a Patient With Guillain-Barre Syndrome Undergoing Lower-Segment Cesarean Section (LSCS): A Case Report

**DOI:** 10.7759/cureus.77263

**Published:** 2025-01-10

**Authors:** TC Arun, Sudeep Takoor, Sarita Ramchandani, Swati Vijapurkar, Gade Sandeep

**Affiliations:** 1 Anesthesiology and Critical Care, All India Institute of Medical Sciences, Raipur, Raipur, IND; 2 Anesthesiology, All India Institute of Medical Sciences, Raipur, Raipur, IND; 3 Anesthesiology, Critical Care, and Pain Medicine, All India Institute of Medical Sciences, Raipur, Raipur, IND; 4 Cardiac Anesthesia: Anesthesiology, All India Institute of Medical Sciences, Raipur, Raipur, IND

**Keywords:** acute flaccid muscle weakness, anaesthesiology, autoimmune, gastrointestinal infection, pregnancy

## Abstract

Anesthesia management for a pregnant patient with Guillain-Barré syndrome (GBS) requires careful planning to ensure the safety of both the mother and fetus while addressing its specific physiological challenges. Management should involve a coordinated team of anesthesiologists, obstetricians, neurologists, and intensivists to optimize outcomes for the mother and fetus. There appear to be no specific case reports that directly document the use of supraglottic airway devices (SGADs) in managing patients with GBS. GBS is a rare, acute autoimmune disorder in which the body’s immune system attacks the peripheral nervous system, leading to muscle weakness and potentially life-threatening complications like respiratory failure. GBS during pregnancy is particularly challenging due to its rarity and the need to balance maternal and fetal health. GBS is often preceded by infections (e.g., Campylobacter jejuni, cytomegalovirus) or other immune system triggers. Here, we describe the successful anesthetic management with SGAD of a 28-year-old pregnant woman with GBS following a gastrointestinal infection.

## Introduction

Guillain-Barré syndrome (GBS) is an autoimmune condition where the immune system attacks the nerves. The condition may be triggered by an acute viral or bacterial infection or a vaccine where a person's immune system attacks peripheral nerves [1]. Symptoms begin as tingling and weakness in the legs and feet that spread to the upper body, characterized by progressive ascending polyneuropathy. Molecular mimicry between epitopes found on gangliosides found on Schwann cell membranes and the cell walls of microorganisms is thought to be the pathogenesis of GBS [2]. It is a rare occurrence in pregnancy. This type of polyneuropathy has the potential to cause severe respiratory depression, which is complicated by pregnancy. Autonomic dysfunction in GBS can cause blood pressure instability, which increases the risk of obstetric complications like preeclampsia or uteroplacental insufficiency [3]. GBS occurring in the first or second trimester is rare but may pose challenges due to prolonged recovery times. When GBS develops in the third trimester, obstetricians may need to decide between delaying delivery for maternal stabilization or early delivery based on fetal well-being [4,5]. Here, we report the case of a 28-year-old female with GBS following a gastrointestinal infection, and the anesthetic management for elective lower-segment cesarean section (LSCS) using a supraglottic airway device (SGAD).

## Case presentation

A 28-year-old primigravida, 158 cm and 62 kg at 28 weeks of gestation, with an estimated date of delivery of 25/05/2024 presented to the hospital with complaints of loose stools and vomiting. The patient’s past medical and surgery history was insignificant, and her antenatal history until this point was uneventful. With the provisional diagnosis of acute gastroenteritis, the patient was started on intravenous fluids and antibiotics. The laboratory investigations were all within acceptable limits, except for stool culture, which was positive for campylobacter.

While she was on the treatment, the patient slowly started developing weakness in her lower limbs, which slowly started to ascend to her upper limbs over two days. The patient was evaluated for GBS, and the diagnosis was confirmed by positive anti-ganglioside subtype M1 (anti-GM1) antibodies. The patient also tested positive for COVID-19; this was confirmed by real-time reverse transcriptase polymerase chain reaction (RT-PCR) to be severe acute respiratory syndrome coronavirus 2 (SARS‑CoV‑2). Gradually, the patient developed respiratory distress, and the patient was intubated given the risk of aspiration. The patient was mechanically ventilated and intravenous immunoglobulins were started. The patient’s respiratory mechanics gradually improved, and she was extubated after 15 days. Tablet pyridostigmine was started at a dose of 300 mg per day. Maternal neurological assessment was carried out throughout the antepartum period while fetal ultrasound was performed to assess the growth of the fetus.

The patient was planned for elective cesarean delivery after 36 weeks of gestation for fetal lung maturity. Fetal ultrasonography at 36 weeks of gestation showed an estimated fetal weight of 2.2 kilograms (Kg), longitudinal lie, and cephalic presentation. No fetal anomalies were detected on the ultrasound. At the time of preoperative assessment, the motor assessment of the patient is shown in Table 1.

**Table 1:** Preoperative motor assessment of the patient

1. Motor power	Right	Left
a. Upper limb	1/5	1/5
b. Lower limb	2/5	2/5
2. Tone		
a. Upper limb	Decreased (Hypotonia)	Decreased (Hypotonia)
b. Lower limb	Decreased (Hypotonia)	Decreased (Hypotonia)
3. Reflexes		
a. Biceps jerk	Hyporeflexia (Grade 1)	Hyporeflexia (Grade 1)
b. Knee jerk	Hyporeflexia (Grade 1)	Hyporeflexia (Grade 1)
c. Plantar reflex	Normal (Grade 2)	Normal (Grade 2)

A complete neurological and cardiovascular assessment was performed. The preoperative vitals of the patient were a heart rate of 90 beats per minute, blood pressure of 110/68 mmHg, and oxygen saturation of 98% on room air. Fasting guidelines of eight hours for solids and two hours for clear liquids were advised and the patient was cleared for surgery under the American Society of Anaesthesiologists Physical Status III. High-risk consent was obtained from the patient's attendees given the requirement of prolonged ventilation and the need for tracheostomy in case of failure to wean the patient.

On the day of surgery, Inj pantoprazole 40 mg was administered. Standard American Society of Anaesthesiologists (ASA) monitors were attached, which included an electrocardiogram, non-invasive blood pressure, pulse oximetry, temperature, and end-tidal carbon dioxide. The choice of anesthesia was general anesthesia with a second-generation SGAD without neuromuscular blockade. The patient was induced with Inj fentanyl 40 micrograms and a sleep dose of propofol (60 milligrams). After the loss of verbal commands, a second-generation SGAD (igel size 3) was inserted. The position of the airway was checked with a leak of less than 5% of the preset tidal volume on the ventilator in the volume control mode and a suction catheter was inserted into the gastric port. Anesthesia was maintained with an infusion of propofol at 75 mcg/kg/min, and oxygen and nitrous oxide at 50% and 50% respectively. Lung protective ventilation strategies were used with tidal volume at 6-8 mL/kg, and a respiratory rate of 12-14 to maintain an end-tidal carbon dioxide concentration between 35 and 45 mmHg.

A healthy, live male child was delivered with a birth weight of 2.3 kg. Post-delivery of the child, an additional bolus of fentanyl 30 micrograms was administered. Intraoperative blood loss, uterine tone, and urine output were closely monitored. The patient produced a urine output of 300 mL while 1500 mL of ringer lactate was administered. Blood loss was around 550 mL. The patient maintained stable hemodynamics throughout the procedure, which lasted for 55 minutes. At the time of skin closure, propofol infusion was stopped and a bilateral landmark-guided transversus abdominis plane (TAP) block was given using 0.25% bupivacaine, 15 mL on each side for analgesia. With the return of spontaneous respiratory efforts, a thorough suction of the oral cavity was performed and SGAD was removed. The patient was then shifted to the post-anesthesia care unit (PACU) equipped with emergency drugs and an airway cart where the patient was monitored for one hour. After an uneventful stay in the recovery room, the patient was then shifted to the critical care unit (CCU) for observation. On postoperative day 1, the patient with stable hemodynamics and no episodes of vomiting or regurgitation overnight was shifted to the ward for further management. The baby was shifted to the neonatal intensive care unit given low birth weight. The child was observed for 72 hours after which the the child was shifted to the mother’s side. The motor power of the mother did not improve significantly in the first week following the delivery. The patient was discharged on the postoperative day 8. On follow-up after three months, the power in both upper and lower limbs improved to 4/5.

## Discussion

GBS is a neurological disorder in which the body's immune system mistakenly attacks the peripheral nerves. It typically causes progressive muscle weakness, areflexia, and in severe cases, paralysis. The progression of weakness in two or more limbs should be less than four weeks [6]. GBS can progress rapidly and is considered a medical emergency. It is the most common cause of acute flaccid paralysis. Though more common in the elderly, all age groups can be affected.

The diagnosis of GBS can be challenging, as it presents with unclear symptoms of weakness, backaches, and paraesthesias. Patients in whom GBS is suspected must be observed in case life-threatening bulbar dysfunction or paralysis evolves rapidly. Most patients report antecedent infection (e.g. upper respiratory tract infection or diarrhea, classically due to Campylobacter jejuni) in the previous six weeks [7]. It is also associated with the administration of swine influenza vaccines [8]. Treatment involves supportive care such as monitoring respiratory function and physical therapy. First-line treatment usually involves intravenous immunoglobulin (IVIG). Plasma exchange (plasmapheresis) helps remove antibodies that attack nerves.

GBS, being rare in pregnancy, has an incidence between 1.2 and 1.9 cases per 100,000 females and carries a high maternal risk. There is an increased requirement for ventilatory support and raised maternal mortality. A retrospective study by Shri Ram Sharma et al. found that acute demyelinating polyneuropathy (AIDP) was the predominant form of GBS [9]. In the majority of the patients, weakness started in the lower limbs. The third trimester and first two weeks have the highest risk of contracting GBS.

Difficulty in coughing out secretions, dysphasia, and inability to protect the airway are manifestations of bulbar palsy. These have to be checked preoperatively since they increase the risk of aspiration [10]. Ensuring appropriate fasting times to minimize the risk of aspiration, particularly in patients with weakened swallowing or bulbar dysfunction, is essential. Assessment of the extent of neuromuscular involvement, particularly respiratory muscle weakness and autonomic dysfunction, is imperative. Diaphragmatic involvement may necessitate ventilatory support. There are increased chances of hypotension due to hypovolemia and autonomic dysfunction and more than anticipated blood loss may occur during delivery.

The central neuraxial block is usually avoided since delivery of pregnant GBS under epidural anesthesia has reported worsening neurological status [11]. Exaggerated hemodynamic alterations that can result from spinal/epidural techniques may also be a reason to avoid neuraxial techniques. General anesthesia with endotracheal intubation is widely used in such cases. Muscle relaxants have variable responses and chances of hyperkalemia with succinylcholine have been reported [12]. Endotracheal intubation with muscle relaxation may cause muscle weakness, which might lead to prolonged postoperative mechanical ventilation. In a similar case report by Sajjan Prashant Shivaraj et al. administered general anesthesia with endotracheal intubation and used rocuronium as the muscle relaxant for the anesthetic management of a patient with GBS during an emergency cesarean section [13]. In our case, we used a second-generation SGAD by which we could avoid the use of muscle relaxants. We could successfully wean the patient off the ventilator in the immediate postoperative period.

## Conclusions

The anesthetic management in patients with GBS requires meticulous planning due to potential respiratory, autonomic, and neuromuscular complications. The choice of anesthesia should be guided by the patient’s clinical status, respiratory function, and surgical requirements. A safe perioperative patient outcome can be achieved with careful assessment and documentation of the patient's baseline neurological status, a comprehensive medication history, and prompt patient discussion of the advantages and disadvantages of various procedures. Second-generation SGAD may be considered an alternative airway strategy in GBS patients to avoid the risk of prolonged mechanical ventilation associated with endotracheal intubation and neuromuscular blockade.
